# Efficient Removal of Methylene Blue and Ciprofloxacin from Aqueous Solution Using Flower-like, Nanostructured ZnO Coating under UV Irradiation

**DOI:** 10.3390/nano12132193

**Published:** 2022-06-26

**Authors:** Vasile Tiron, Mihai Alexandru Ciolan, Georgiana Bulai, Gabriela Mihalache, Florin Daniel Lipsa, Roxana Jijie

**Affiliations:** 1 Research Center on Advanced Materials and Technologies, Department of Exact and Natural Sciences, Institute of Interdisciplinary Research, Alexandru Ioan Cuza University of Iasi, Iasi 700506, Romania; vasile.tiron@uaic.ro (V.T.); mihai.ciolan@uaic.ro (M.A.C.); 2 Integrated Center of Environmental Science Studies in the North-Eastern Development Region (CERNESIM), Department of Exact and Natural Sciences, Institute of Interdisciplinary Research, Alexandru Ioan Cuza University of Iasi, Iasi 700506, Romania; georgiana.bulai@uaic.ro (G.B.); gabriela.mihalache@uaic.ro (G.M.); 3 Department of Horticultural Technologies, “Ion Ionescu de la Brad” University of Life Sciences, M. Sadoveanu Alley, No. 3, Iasi 700490, Romania; 4 Faculty of Agriculture, “Ion Ionescu de la Brad” University of Life Sciences, M. Sadoveanu Alley, No. 3, Iasi 700490, Romania; flipsa@uaiasi.ro; 5 Department of Exact and Natural Sciences, Institute of Interdisciplinary Research, Alexandru Ioan Cuza University of Iasi, Iasi 700506, Romania

**Keywords:** Thermionic Vacuum Arc, zinc-oxide coating, flower-like architectures, photocatalysis, methylene blue, ciprofloxacin

## Abstract

Flower-like ZnO architectures assembled with many nanorods were successfully synthesized through Thermionic Vacuum Arc, operated both in direct current (DC-TVA) and a pulsed mode (PTVA), and coupled with annealing in an oxygen atmosphere. The prepared coatings were analysed by scanning-electron microscopy with energy-dispersive X-ray-spectroscopy (SEM-EDX), X-ray-diffraction (XRD), and photoluminescence (PL) measurements. By simply modifying the TVA operation mode, the morphology and uniformity of ZnO nanorods can be tuned. The photocatalytic performance of synthesized nanostructured ZnO coatings was measured by the degradation of methylene-blue (MB) dye and ciprofloxacin (Cipro) antibiotic. The ZnO (PTVA) showed enhancing results regarding the photodegradation of target contaminants. About 96% of MB molecules were removed within 60 min of UV irradiation, with a rate constant of 0.058 min^−1^, which is almost nine times higher than the value of ZnO (DC-TVA). As well, ZnO (PTVA) presented superior photocatalytic activity towards the decomposition of Cipro, after 240 min of irradiation, yielding 96% degradation efficiency. Moreover, the agar-well diffusion assay performance against both Gram-positive and Gram-negative bacteria confirms the degradation of antibiotic molecules by the UV/ZnO (PTVA) approach, without the formation of secondary hazardous products during the photocatalysis process. Repeated cyclic usage of coatings revealed excellent reusability and operational stability.

## 1. Introduction

In recent years, the frequent detection of organic dyes and antibiotics in water bodies has attracted much concern, owing to the fact that they may have a negative impact on aquatic organisms and human health. The occurrence of quinolone residues (Qs) and azo dyes was reported all around the world, which is not surprising, given that over 0.7 million tons of azo dyes are annually synthesized [1], and the quinolone consumption in the EU/EEA community has been estimated in the range of 2.86 defined daily dose (DDD) per 1000 inhabitants per day in Bulgaria (the highest) and 0.35 per 1000 inhabitants per day in Norway (the lowest) in 2017 [2]. Ciprofloxacin (Cipro), one of the most prescribed Qs used to prevent and treat infectious diseases, often appears in surface water, drinking water, ground water and waste water, ranging from ng/L to mg/L [3,4,5,6,7]. Similarly, methylene blue (MB), a blue cationic thiazine hazardous dye, is detected in the environment after being released in water bodies via effluents from textile, plastic, cosmetic, paper and pharmaceutical industries [8]. Exact data on the MB amount discharged in the environment are not available, but it is assumed that 10–15% of dyes are lost in the effluents during different stages of manufacture [9].

Therefore, much effort has been made to remove or convert such organic pollutants into less harmful by-products [10,11,12,13]. Many physical, chemical and biological methods have been explored to purify and, thereby, selectively remove these toxic contaminants present in water bodies [14]. These include adsorption, membrane filtration, Fenton oxidation, microwave catalysis, electrochemical oxidation and photocatalysis [15,16,17]. Among these various strategies of decontamination, the *in situ* production of charge carriers and powerful reactive species, as the result of the interaction of the semiconductor photocatalysts with a natural or artificial light source, has proven to be one of the most effective greenways to degrade the various organic molecules in aqueous solutions [18,19,20]. Titanium dioxide (TiO_2_) and zinc oxide (ZnO) have attracted as much attention as metal-oxide semiconductor-driven photocatalysis, due to their environmental friendliness, high-degradation efficiencies, easy preparation methods and nontoxic nature. In particular, ZnO appears to be a cost-effective and suitable alternative to the commonly used TiO_2_ photocatalyst [21]. However, there are still some limitations of pure ZnO, such as the large bandgap (~3.37 eV, which corresponds to a wavelength absorption edge ~368 nm) that hinders its application under solar irradiation, the fast electron-hole recombination and limited reusability, which drastically diminish its photocatalytic performance. Therefore, serious efforts have been made to address these drawbacks and enrich the photodegradation activity of ZnO. An improvement in the photocatalytic behaviour of ZnO has been obtained by doping with foreign ions [22,23,24], anchoring with porphyrins [25] or coupling with graphene [26,27] and conducting polymers [28,29]. The photoactivity of ZnO is not only determined by its chemical composition, but also by its morphology, the electronic structure and its crystalline degree and phase [30,31]. Furthermore, the photocatalytic properties are strongly correlated with their specific surface areas, since a photocatalyst with a large specific surface exposes more active sites and exhibits a high photocatalytic performance. The enlarged specific surface area is not only favourable for enhancing contact of the catalyst with the electrolyte, but it is also beneficial for charge and mass transport, contributing to the significant enhancement of the photocatalytic performance [32]. Reducing the particle size of a photocatalyst may decrease the charge recombination probability because it shortens the diffusion pathway of the charge carriers. Nanostructured materials offer the opportunity to minimize the distances and time over which charge carriers have to survive and be transported towards the surface, where they will drive photocatalytic reactions [33].

Besides optical bandgap and morphology, photocatalytic performance is strongly related to electrical conductivity, which in turn can significantly affect the hole and electron transfer. It is well known that electrical conductivity varies with the crystal structure and stoichiometric form of the photocatalyst, since the bulk/surface defects of the photocatalyst usually act as recombination centers for photoexcited electrons and holes. Therefore, increasing the crystallinity degree of photocatalysts can reduce the probability of the charge recombination between photo-generated electrons and holes, resulting in increases in the lifetime and mobility of charge carriers [34]. Even if the photo-generated electrons and holes possess sufficient potential for photo-degradation reactions, the lack of active sites on the photocatalyst surface leads to charge-recombination reactions. Co-catalysts, such as Pt, NiO and RuO_2_, are usually loaded on the photocatalyst surface in order to confine the photo-excited charge carriers to the surface, thus avoiding the charge recombination and introducing new active sites [35]. Another strategy to reduce the recombination of the photogenerated charges is to separate the electrons and holes by using different facets of the crystal photocatalyst [36] or by using a one-dimensional nanostructured photocatalyst [37]. When using one-dimensional nanostructures, such as nanorods or nanowires, photo-excited electrons migrate along the nanostructure axis towards the tip, while the holes migrate towards the sides, leading to effective separation of the photogenerated charges.

Synthesis approaches play crucial roles in determining the bulk and surface properties as well as the performance of photocatalyst materials. Over the past few years, various physical and chemical methods have been employed for the synthesis of materials based on ZnO, including simple precipitation [38], sol–gel [39], hydrothermal [40], electrochemical [41] and microwave-assisted-deposition techniques [42]. Even though these approaches are the most common routes for the preparation of ZnO, they are expensive and time-consuming, plus they require the use of toxic chemical compounds or organic solvents as reducing agents, which are all limitations that can be overcome with Physical Vapour Deposition (PVD) techniques. For this reason, increasing attention has been put on eco-friendly deposition approaches that are assisted by plasma processes. A wide range of plasma-processing thin-film technologies are available, such as pulsed laser deposition, sputtering techniques and the termionic vacuum arc. The coating’s quality is linked to the experiment setup and process parameters (e.g., ion flux and energy of vaporised material). For example, the TVA is a gas-free plasma source with ion energy and flux that can be easily controlled by operating parameters [43]. Apart from the fact that the TVA-deposition method is an environmentally friendly, time-saving, cost-effective and facile PVD technology, the definite advantages are the high deposition rates, high purity of the thin films and good adhesion of the coating to the substrate [44]. In addition, by using ZnO thin films [45] or by loading the ZnO particles on a suitable substrate [23,46,47], the need for post-treatment steps to recover or remove the suspended particles from the treated water is avoided.

In the present study, the TVA-deposition method, operated both in DC and a pulsed mode, was used to synthesize Zn coatings, which were subsequently annealed at 800 °C in an oxygen atmosphere for 6 h to obtain the nanostructured ZnO coatings. The synthesized photocatalysts were characterized by a scanning-electron microscopy with energy-dispersive X-ray-spectroscopy (SEM-EDX), X-ray-diffraction (XRD) and photoluminescence (PL) measurements. Initially, the photocatalytic-degradation efficiency of the ZnO coatings was evaluated by assessing the removal of methylene-blue dye (MB) in water under UV irradiation. Then, due to the greater photocatalytic activity of ZnO (PTVA), this was used as a photocatalyst for the mineralization of ciprofloxacin (Cipro) in aqueous solution. Furthermore, to prove the effective removal of Cipro from water, the agar-well-diffusion assay against both Gram-positive and Gram-negative bacteria was performed. The reusability of nanostructured ZnO samples was also investigated by recording the photocatalytic activity over four consecutive cycles of 240 min under UV irradiation. To the best of our knowledge, the deposition of Zn-metallic coatings by TVA working in a pulsed mode and the examination of flower-like, nanostructured ZnO-coating photocatalytic activity, under UV irradiation using Cipro antibiotic as a model contaminant, have not been reported so far.

## 2. Materials and Methods

### 2.1. Photocatalyst Synthesis

TVA is a gas-free metal-vapour plasma source, characterized by highly energetic metal ions and high deposition rate. Briefly, TVA discharge takes place between two electrodes (anode and cathode), in the vapours of the anode material, vapours that are produced under the intense electron bombardment. An overview of TVA operation principle and applications is given by Vladoiu et al. in a recent review [48]. Usually, the discharge cathode consists of a loop-shaped tungsten filament, surrounded by a Wehnelt cylinder used to focus thermo-electrons, while the anode is usually shaped as a crucible (cylindrical shaped), made from materials with high melting point (W, C, TiB), which contains the material to be evaporated (small pieces of Zn, in this case). The anode is highly positively biased (from few hundreds of V up to several kV), while the cathode is grounded and acts as an electron gun when is heated. Under the electron-beam bombardment, the anode material first begins to melt and afterwards to boil and evaporate, ensuring a steady state concentration of evaporated atoms in the inter-electrodic space, which meet the necessary conditions for a bright arc plasma ignition in the anode’s vicinity. An important feature of the thermionic arc is that the plasma bulk is highly positive (hundreds of V to 1 kV), so that the metal ions will be accelerated towards the growing film with energy corresponding to the potential drop between the plasma potential and substrate potential [49,50]. An extensive description of experimental setup (excepting vacuum vessel size) and plasma diagnostics techniques are given in our previous paper [44]. In this work, all the experiments were performed in an ultra-high vacuum stainless-steel chamber with cylindrical shape (30 cm in diameter and 50 cm height). Prior to arc ignition, the chamber was depressurized to an ultimate pressure of 10^−4^ Pa using a pumping system consisting of a turbo-molecular pump and a dry pump. During TVA discharge, due to metal vapours, the pressure inside the chamber increases up to 10^−3^ Pa. Several Zn coatings were deposited on silicon (Si) substrates, by operating TVA discharge both in direct current (DC-TVA) and a pulsed mode (PTVA). Our previous studies have shown that as compared to conventional DC–TVA, PTVA can generate metal-vapour plasma characterized by higher energetic ions and a much higher ionization degree [51]. Therefore, it is expected that the properties on Zn coatings to be related to the plasma conditions, which, in turn, depend on the operation mode of TVA discharge. In the case of DC-TVA plasma, the discharge current was set to 300 mA, while the discharge voltage was set to +400 V, using a DC power supply. The PTVA discharge was operated with amplitude voltage U = 1 kV, peak current I_p_ = 1.5 A, pulse duration τ = 200 µs and repetition frequency ν = 1 kHz, using a home-made pulse generator. The average power during PTVA operation was 280 W. In both operation modes, filament current (I_f_) was set to 25 A, while the distance between filament and anode (d_a-f_) was set to 3 cm. The Si substrates were fixed on an electrically grounded substrate holder, axially positioned at 20 cm above the anode. In both cases, the deposition time was set to 10 min, and the substrates were unintentionally heated during deposition process. The as-deposited Zn coatings were then annealed at 800 °C for 6 h under an oxygen atmosphere to obtain ZnO nanostructures. The annealing process was carried out in a high vacuum stainless-steel chamber using a programmable heater system. Prior to annealing process, the chamber was depressurized to an ultimate vacuum of 10^−4^ Pa using a pumping system consisting of a turbo-molecular pump and a rotary pump. The annealing process was conducted at a designed heating and cooling rate, in an oxygen atmosphere at pressure of 1 Pa. The oxygen gas was introduced into the chamber with a constant flow rate of 10 sccm (standard cubic centimetres per minute). The samples (Zn coatings deposited onto Si substrates) were heated from room temperature (RT = 20 °C) to 800 °C, with a heating rate of 3.2 °C/min, and after 6 h of annealing were cooled down to RT with a rate of 1.6 °C/min.

### 2.2. Plasma Diagnosis

During DC-TVA operation, the discharge voltage and current were directly monitored from power-supply display, while during PTVA operation, the voltage and current waveforms were recorded by a digital oscilloscope using a high-voltage probe (TesTec, 1:100) and a current probe (Pearson, 1 V/1 A). In both operation modes, the space-and-time evolution of the plasma potential has been recorded using an emissive probe system working in the so-called “saturated floating-potential regime” [52]. A detailed description of plasma-potential-measurement technique was reported in one of our previous works [53]. In this work, plasma-potential measurements have been performed along the TVA discharge axis, between Wehnelt cylinder and top side of the chamber wall (35 cm above the anode), using a step size of 5 mm.

### 2.3. Materials Characterization

The morphology and composition of the as-deposited Zn and annealed (ZnO) coatings were investigated *via* field-emission scanning-electron microscopy (Hitachi S-3400N, Hitachi Science Systems, Tokyo, Japan) equipped with an energy-dispersive X-ray spectrometer (EDX). The structural and phase analyses of the coatings were performed using X-ray diffraction (XRD), in Bragg–Brentano configuration, via Shimadzu LabX XRD-6000 diffractometer (supplied by Shimadzu Corporation, Kyoto, Japan), equipped with a CuKα radiation source (λ = 1.54059 Å). The photoluminescence (PL) measurements were performed at room temperature using SLM 8000 spectrofluorometer (SLM Instruments, Urbana, IL, USA), with an excitation wavelength of 330 nm.

### 2.4. Photocatalytic Activity Measurements

The photocatalytic performance of nanostructured ZnO coatings, under UV irradiation, was investigated by using methylene blue (MB, 03978-250ML, Sigma Aldrich, Darmstadt, Germany) and ciprofloxacin (Cipro, Novo mesto, Slovenia) antibiotic as model contaminants. The Cipro liquid form was purchased from a local pharmacy. The manufacturer brand remains anonymous to avoid a possible conflict of interest. The product was selected in order to explore a scenario similar to a real case. The photocatalytic activity measurements have been carried out in a 50 mm × 17 mm glass Petri dish illuminated by a horizontally placed UV lamp (Philips, Poland, 1 W/m^2^, with main emission wavelength at 253.7 nm), at 5 cm above the sample, inside of a self-designed box covered with an aluminium foil. The power density of the incident light was measured using a UV-Optometer (G187079, SUSS Microtek, Eindhoven, The Netherlands). Prior to the light irradiation, the aqueous solution of MB (47 μM) or Cipro (0.015 μM) with the photocatalyst sample (nanostructured ZnO coating deposited onto 2 × 2 cm^2^ Si wafer) was kept in contact in the dark for 30 min in order to achieve the adsorption-desorption equilibrium. The antibiotic concentration was selected according to its occurrence in wastewater and water resources [54]. At the pre-set time intervals, solution was collected and the changes in the contaminant concentration were analysed with Evolution 300 UV-Vis spectrophotometer (Thermo Fisher Scientific, Madison, WI, USA). The degradation of the organic contaminants was followed by measuring the decay of the absorption intensity at their λ_max_ = 664 nm (MB) and 277 nm (Cipro) as a function of irradiation time. The concentrations of treated dye and antibiotic solutions were evaluated from constructed calibration curves, as shown in Figure S1 (Supplementary Materials). The removal percentage (%) of pollutants was calculated using Equation (1), where C_0_ is the MB/Cipro initial concentration after 30 min adsorption in dark, and C_t_ is the dye/antibiotic concentration after a certain irradiation time.

(1) $$\%\mathrm{Removal}=(1-\frac{C_{t}}{C_{0}})\times 100\%$$

The degradation rate constant (k) values were determined from slopes of the graphs by plotting–ln(C_t_/C_0_) versus irradiation time. In addition, the apparent quantum yield (AQY) of the photocatalytic process were estimated using Equation (2), following the calculation details given by Bora et al. [55].

(2) $$\mathrm{AQY}(\%)=\frac{2n_{\mathrm{MB}}N_{A}\mathrm{hc}}{\mathrm{PS}\lambda t}\times 100\%$$

where n_MB_ (mol) is the amount of methylene blue mineralized in 1800 s, N_A_ (mol^−1^) is Avogadro’s constant, h (J·s) is Planck’s constant, c (m·s^−1^) is speed of light, P (W/m^2^) is the power density of the incident light, S (m^2^) is the photocatalyst area, λ (m) is the wavelength of the incident monochromatic light and t (s) is the exposure time.

The stability of synthesized thin film photocatalyst was investigated over four consecutive cycles of 240 min under UV irradiation.

### 2.5. Agar Well Diffusion Assay

In order to assess the efficient removal of ciprofloxacin from aqueous solution by nanostructured ZnO (PTVA) coating under UV irradiation, the well-diffusion assay was performed against both bacterial strains, Escherichia coli ATCC 8739 (Gram-negative bacilli, Microbiologics, MN, USA) and Staphylococcus aureus ATCC 25923 (Gram-positive cocci, Microbiologics, MN, USA) following the protocol described by Dwivedi et al. [56]. Briefly, both bacteria were inoculated and incubated overnight at 37 °C and 160 rpm in 10 mL Muller–Hinton broth. The optical density measured at 600 nm (OD600) of the cultures was adjusted to 0.1 (approx. 3.5 × 10^8^ CFU/mL) after incubation, and 200 µL of each inoculum was evenly spread on Muller–Hinton agar in Petri dishes. Using a sterile 1 mL micropipette tip, wells of approximately 10 mm diameter were made in the agar layer (4 per Petri dish). Then, 100 µL of control (Cipro without UV and ZnO coatings) and treated solutions (10, 30, 120, 240 min) with UV in absence or presence of photocatalyst film were poured into each well. The clear zone diameter (mm) was measured after plates were incubated overnight at 37°C, as illustrated in Figure S2 (Supplementary Materials). To ensure the consistence of our results, the tests were performed in triplicates.

## 3. Results and Discussions

### 3.1. Plasma Characterization

A deep understanding of the pulsed TVA discharge behaviour is necessary to control the deposition process, process stability and to improve the coating performance. Plasma-diagnosis results allow for better selection of the experimental setup and process parameters, to get the maximum benefit from this technique. The plasma potential and, consequently, the ion flux and energy of vaporised material is of great importance when investigate the correlation between plasma properties and coating structure. In this work, to gain a better understanding of the fundamental mechanisms governing a pulsed TVA process and to investigate their implications on the coating growth, the discharge current-voltage behavior and plasma-potential distribution were measured and discussed.

**Figure 1:**
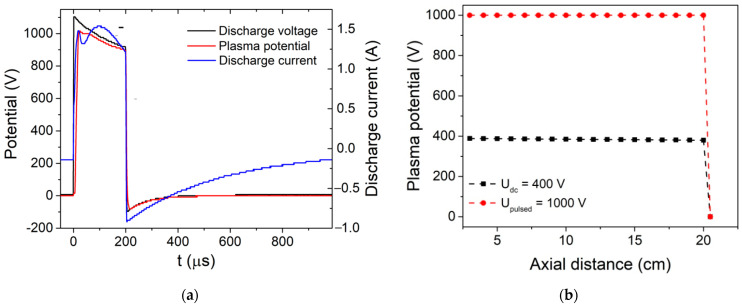
Temporal evolutions of (**a**) discharge voltage, discharge current and plasma potential measured during PTVA with discharge voltage U = 1 kV, pulse duration τ = 200 µs and repetition frequency ν = 1 kV. (**b**) Axial distribution of peak plasma potential measured between Wehnelt cylinder and port substrate during DC-TVA (U = 400 V, I = 300 mA) and PTVA discharges.

Figure 1a shows the temporal evolutions of the discharge voltage, discharge current and plasma potential measured at the substrate position (20 cm above the anode) during PTVA discharge with U = 1 kV, pulse duration τ = 200 µs and repetition frequency ν = 1 kHz. The temporal evolution of the plasma potential, measured at the substrate position by an emissive probe, follows closely the discharge-voltage waveform, having quite similar values. During the on-pulse period, the peak current reaches a value of 1.5 A, which is five times higher than discharge current during DC-TVA operation. This translates in a much higher plasma density and ion flux towards the substrate in the case of PTVA as compared to the DC-TVA discharge.

The fast replication of the discharge current during the PTVA pulse and its time evolution in-between pulses indicates that the afterglow plasma does not die out, as the ions and electrons are still present at the beginning of the subsequent pulse. The axial distribution of plasma potential measured between the Wehnelt cylinder and the port substrate (Figure 1b) shows that in both cases the plasma-potential distributions (measured as peak plasma potential during the PTVA pulse) are uniform, with values very close to the discharge voltage. Plasma-potential measurements (not shown here) performed beyond the substrate position, up to the top side of the chamber wall (35 cm above the anode), reveal also a uniform plasma-potential distribution. This feature is much different from the plasma-potential-distribution data reported before, for Cu and Be TVA plasmas, due to the much lower melting point of Zn (682.7 K versus 1356.6 K and 1560 K, respectively) and lower size (especially, diameter) of the discharge chamber. Due to a very low melting point, the evaporation rate of the Zn is very high and TVA plasma fills the whole chamber, just like in the case of a glow discharge. Due to very large gradient of metallic vapours and the open geometry of the discharge electrodes, the plasma species diffuse in almost all directions (radial losses), allowing very large substrates to be coated. It should be noted that the deposition rate, estimated by means of a profilometer, of the Zn coating deposited onto Si substrate positioned axially at 20 cm above the anode, is about 2 µm/min during DC-TVA operation and 4.5 µm/min during PTVA discharge. In addition, the plasma-potential-distribution measurements reveal that Zn ions are accelerated towards a grounded substrate with energy corresponding to the full-arc voltage (up to 1 kV during PTVA operations).

### 3.2. Surface Morphology

**Figure 2:**
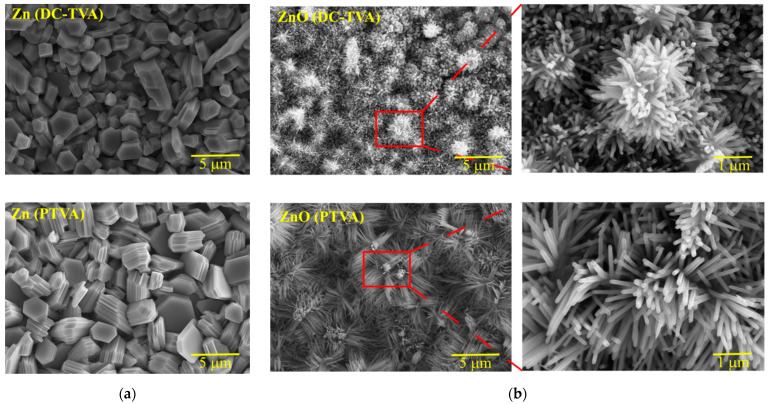
SEM images of the as-deposited Zn coatings (**a**) by DC-TVA and PTVA, and the corresponding ZnO nanostructures (**b**) obtained after annealing in an oxygen atmosphere.

Figure 2 shows SEM images of the as-deposited Zn coatings by DC-TVA and PTVA and corresponding flower-like ZnO structures obtained after thermal treatment in an oxygen atmosphere at 800 °C for 6 h.

The Zn coatings deposited either by DC-TVA or PTVA present an aspect of nanostructures with regular and irregular shapes (hexagonal drums and truncated hexagonal pyramids). The Zn nanostructures obtained by DC-TVA have a compact structure, while those obtained by PTVA have a lamellar structure. It seems that Zn nanostructures synthesized by PTVA appear to be made of overlapping hexagonal nanosheets. The pulsed-plasma regime and higher kinetic energy of the Zn ions gained during PTVA discharge lead to the growth of self-assembly nanostructures. After annealing in an oxygen atmosphere, the resulting ZnO coatings revealed flower-like surface morphologies, consisting of numerous rods with individual diameters around 30–50 nm. For instance, flower-like ZnO architectures have been prepared by ultrasonic and microwave combined technique or hydrothermally using solution-based approaches [57,58,59,60]. In addition, an increase in the density and length of the ZnO nanorods has been observed for the coating obtained by PTVA. In contrast with previous reports, where the length of nanorods has been controlled by altering the precursor concentration [61] and the pre-annealing temperature of the ZnO seed layer [62], the change of TVA-operation mode enabled us to produce ZnO nanorods with tunable morphology. 

Among all the materials, the ZnO has the richest variety of nanostructure shapes, such as tower-, tube- and flower-like morphologies [63,64,65], nanotubes [66], nanowires [67], hollow spheres [68], nanohelices [69], nanorods [70], nanobelts [71] and nanoplates [72]. The ability to be synthesized in wide range of novel structures resides from an important characteristic of ZnO, such as its polar surfaces. Structurally, ZnO has a hexagonal crystal structure, with three types of growth direction, corresponding to a polar surface ({0001}) and two non-polar surfaces ({2110} and {0110}). The relative surface activities of various growth facets determine the anisotropic growth rates and the morphology of ZnO nanostructures. ZnO synthesized by PTVA shows a typical growth morphology of 1D nanostructures (nanorods), where the growing structures tend to maximize the areas of the non-polar facets ({2110} and {0110}) and to minimize the area of the polar facet {0001}, which is stable and has higher energy than the non-polar facets. The nanorod’s growth direction seems to be affected by the geometrical shape and crystallographic structure of the Zn nanostructures. The nanorods grow along {0001} facet (c-axis), in the Zn-nanosheet plane, with the direction normal to the side of hexagon.

### 3.3. Structural, Chemical Properties

**Figure 3:**
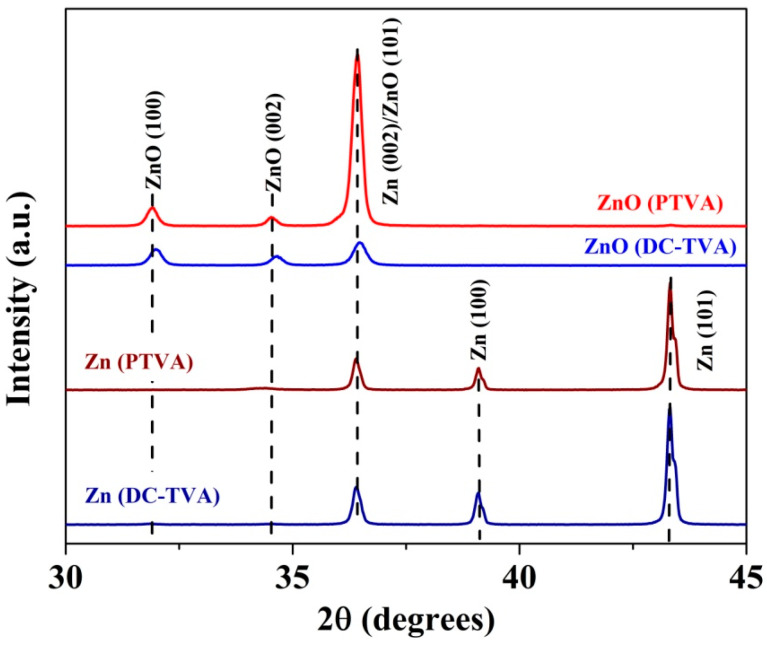
X-ray-diffraction patterns for as-deposited Zn coatings and thermal-annealed coating (ZnO) synthesized by DC-TVA and PTVA.

Figure 3 shows the X-ray-diffraction patterns for as-deposited Zn coatings and the thermal-annealed coating (ZnO) synthesized by DC-TVA and PTVA, respectively. The diffraction patterns of the as-deposited Zn coating deposited by DC-TVA confirm the presence of a Zn phase (according to PDF card no. 870713), with the main diffraction peaks positioned at 36.38°, 39.07° and 43.3°, which are assigned to the (002), (100) and (101) planes of pure Zn, respectively. The diffraction peaks of the as-deposited Zn coating deposited by PTVA are shifted to higher diffraction angles by 0.25°, indicating a tensile stress in the coatings, which could be caused by the grain-boundary shrinkage [73]. The diffraction peaks of Zn coating deposited by DC-TVA have the same intensity as the Zn coating deposited by PTVA, indicating roughly the same crystallinity degree of both coatings. The EDX measurements revealed that the Zn coatings deposited both in DC and pulsed TVA have (97 ± 1) at.% Zn and (3 ± 1) at.% O. The origin of oxygen in the as-deposited coatings can be assigned to residual oxygen from the discharge chamber, desorbed oxygen under intense ion bombardment, from the chamber wall, and to the natural oxidation process of Zn coatings as a result of their exposure to environmental conditions. The average grain size, estimated from the diffraction peaks’ width using Scherrer’s equation [74], is 73.4 nm and 84.2 nm in the case of the as-deposited Zn coating deposited by DC-TVA and PTVA, respectively.

After thermal annealing, the characteristic peaks of the wurtzite ZnO phase is found, while all the characteristic peaks of Zn disappeared, indicating a complete oxidation process of the Zn coatings. This statement is supported by EDX measurements, which revealed, for both coatings, a chemical composition of (51 ± 1) at.% Zn and (49 ± 1) at.% O. The main diffraction peaks positioned at 31.96°, 34.62° and 36.45° are assigned to the (100), (002) and (101) planes of the wurtzite ZnO phase (according to PDF card no. 890511), respectively. As compared to the ZnO coatings deposited by DC-TVA, the X-ray-diffraction pattern of the ZnO coatings deposited by PTVA depicts a better crystalline order, the intensity of ZnO (101) plane being eight times higher. The average grain size of the ZnO nanostructures synthesized by DC-TVA and PTVA is 28.9 nm and 37.6 nm, respectively.

### 3.4. Photoluminescence Spectra

Since the recombination of photoexcited charge carriers produces fluorescence emission, the photoluminescence (PL) spectra may provide information about the recombination rate. Therefore, the high PL-emission intensity is a sign of a high recombination rate of electrons (e^−^) and holes (h^+^), which, in turn, decreases the photoexcited charges available for photocatalytic reactions. On the contrary, a low recombination rate is, thus, expected to produce a low PL emission intensity, meaning that more photoexcited charge carriers are available to participate in the photodegradation reactions. Consequently, to improve the material photocatalytic activity, it is very important to slow down the recombination of the photo-induced charge carriers [75].

**Figure 4:**
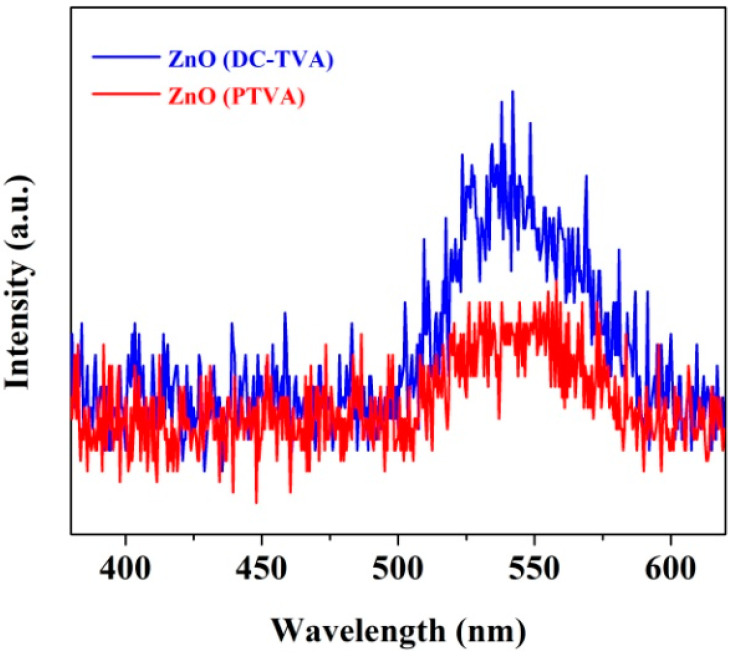
Photoluminescence spectra of ZnO coatings synthesized by DC-TVA and PTVA obtained at room temperature and at the excitation wavelength of 330 nm.

As can be seen in Figure 4, the photoluminescence spectra of ZnO coatings synthesized by DC-TVA and PTVA, display a very low emission band in the range of 495–605 nm. The PL-emission signal could be attributed to the intrinsic defect states in ZnO, such as interstitial oxygen/zinc [76] and oxygen/zinc vacancies [77,78], which act as recombination centers for photoexcited charge carriers. Therefore, the lower intensity of the ZnO (PTVA) PL spectrum corresponds to a lower recombination rate of the electron-hole pairs, thereby enhancing the photocatalytic efficiency.

### 3.5. Evaluation of Photocatalytic Activity

**Figure 5:**
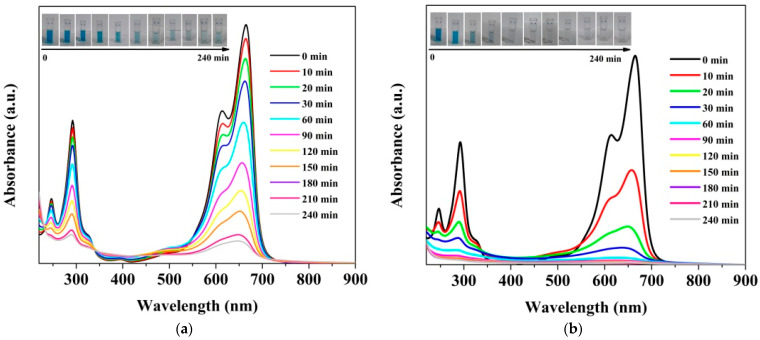
(**a**) UV-Vis-absorption spectra of MB dye as a function of irradiation time acquired in the presence of flower-like, nanostructured ZnO thin film, synthesized by DC-TVA (**a**) and PTVA (**b**), respectively. The inset photos show the colour change of dye solutions during irradiation.

Initially, the photocatalytic efficiencies of the two kinds of ZnO photocatalysts under UV light were evaluated by the degradation of methylene-blue dye, a hazardous chemical found in aqueous emissions from the textile, pharmaceutical and cosmetic industries. Figure 5a,b exhibits changes in absorption spectra of MB solution as a function of irradiation time, in the presence of ZnO synthesized by the DC-TVA and PTVA approaches, respectively. It can be seen that irradiation of MB-dye solution in the presence of photocatalysts leads to a decrease in absorption intensity. The adsorption bands of MB molecules located at 293 nm and 664 nm gradually decrease with an increase in irradiation time and disappear almost completely after 90 min for the flower-like nanostructured ZnO (PTVA) coating.

**Figure 6:**
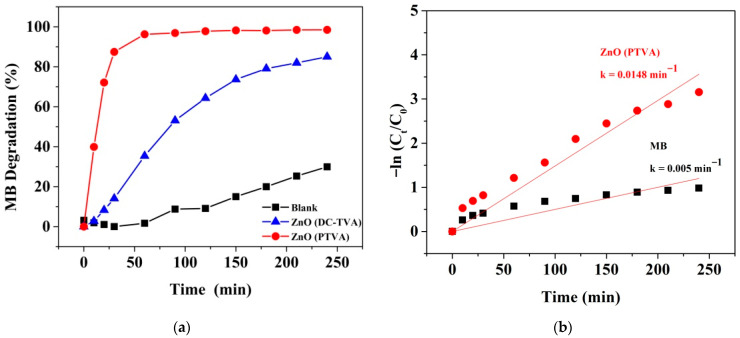
(**a**) The photocatalytic degradation plots of MB dye in absence or presence of nanostructured ZnO samples under UV light. (**b**) Linear kinetic-fitting curves with the degradation-rate constants for the UV-induced dissociation of MB by photocatalysts.

The photocatalytic degradation efficiency of MB dye in the absence or presence of nanostructured ZnO photocatalysts under UV light is shown in Figure 6a. The result indicates that MB is quite stable, with a slight decay in the efficiency in absence of photocatalysts after 240 min of irradiation (ca. 30%). In contrast, the prepared nanostructured ZnO coatings presented enhancing results towards the photocatalytic removal of contaminant, as about 96% of dye removal takes place in the presence of ZnO (PTVA) film under 60 min of UV irradiation, while the photocatalytic degradation efficiency attained by the ZnO (DC-TVA) sample is 35%. The remarkable augmentation of the photocatalytic activity of the nanostructured ZnO coating synthesized by pulsed TVA might be ascribed to the collective contribution of nanostructured morphology and the higher degree of crystallinity, which improve the lifetime of charge carriers and offer more exposed active surface sites, leading to more reactive species and, consequently, to a higher MB photodegradation rate. Similarly, Paisrisarn et al. [79] reported that both the crystallinity and morphology of ZnO nanowires are key physicochemical properties that can be related to the extracellular vesicles’ capture performance. Furthermore, the ZnO (PTVA) sample exhibits a high rate-constant value of 0.058 min^−1^ for the first 60 min of UV irradiation, which is almost nine times the rate of the constant value of the ZnO (DC-TVA) sample (Figure 6b). On the other hand, the apparent quantum yield (AQY), defined as the ratio of the number of MB molecules mineralized in 30 min to the number of incident photons, was calculated for both photocatalysts (Supplementary Materials). The resulting AQYs for photocatalytic degradation of MB using ZnO (PTVA) (26.9%) was almost six times higher than that of ZnO (DC-TVA) (4.3%). These results are in the same range as those found by Bora et al. [55] for the degradation of MB using bare ZnO and gold-nanoparticles-decorated ZnO nanorods under UV irradiation. Owing to the fact that the reactions happen at the interface between the materials’ surface and the organic contaminants, it is expected that the ZnO photocatalytic activity depends on the surface-to-volume ratio of the rods, as well as to the bulk-crystalline degree and phase. It was reported that the formation of 1D nanostructures can efficiently separate electrons and holes spatially, suppressing their recombination rate and greatly enhancing the intrinsic activity of each active site [80]. In addition, using 1D nanostructures, such as nanorods, the excited electrons migrate along the long axis of the nanorod, while the holes migrate to the sides, causing the effective separation of electrons and holes, significantly improving overall the photocatalytic performances [81].

**Figure 7:**
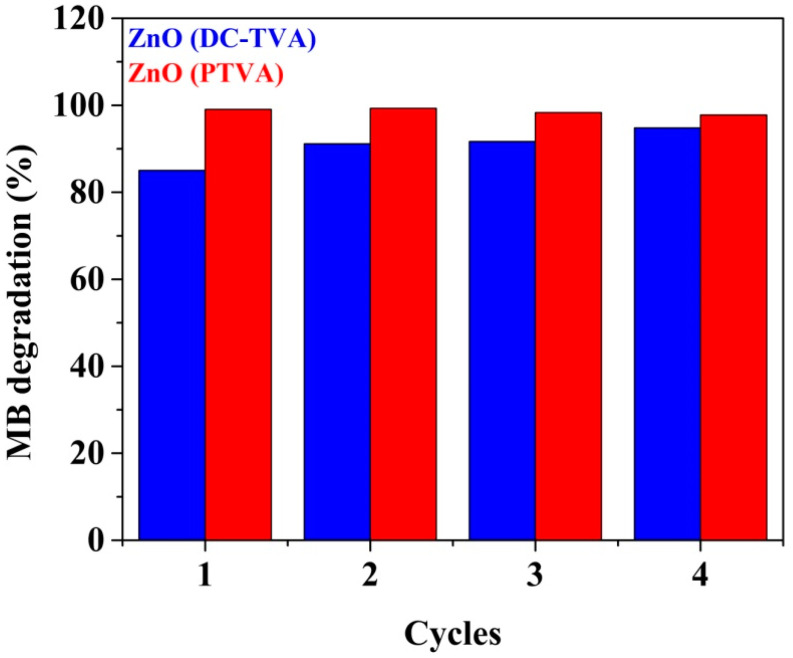
Reuse of ZnO synthesized by DC-TVA and PTVA approaches for the photodegradation of MB for four successive cycles (240 min each run).

One important feature of the synthesized ZnO-based photocatalysts is their reusability: they can give almost the same performance over four consecutive cycles of 240 min under UV irradiation. As shown in Figure 7, a slight decay in the photodegradation efficiency of ZnO coating prepared by PTVA over the cycles (from 99% to 97.8%) has been observed, which might be due to the deactivation of the photocatalyst active sites by adsorbed degradation by-products or due to the conversion of zinc oxide (ZnO) in zinc peroxide (ZnO_2_). On contrary, the ZnO coating synthesized by DC-TVA revealed a slight improvement in its photodegradation performance (from 85% to 94.7%), probably due to the removal/decomposition of organic contaminants (hydrocarbons) adsorbed on the catalyst surface before photocatalytic degradation measurements. Organic compounds act as electron donors for photocatalytic reactions, and a part of the photo-generated holes are used for their decomposition [82].

**Figure 8:**
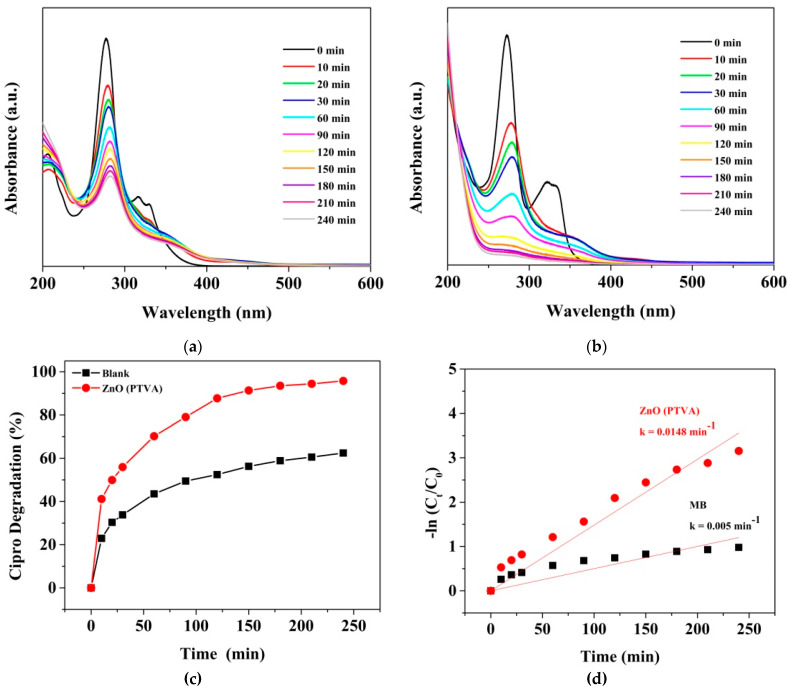
UV-Vis-absorption spectra of Cipro solution as a function of irradiation time acquired in the absence (**a**) and presence (**b**) of ZnO synthesized by PTVA approach. (**c**) Photocatalytic-degradation plots of Cipro in the absence or presence of nanostructured ZnO coating within 240 min of UV irradiation. (**d**) Linear kinetic-fitting curves with the degradation-rate constants for the UV-induced dissociation of Cipro.

To further examine the capability of the ZnO coating synthesized by PTVA for the decontamination of water polluted with pharmaceutical compounds, the prepared photocatalyst has been tested for the degradation of ciprofloxacin, which is the most frequently detected fluoroquinolone antibiotic in wastewater plants and water resources around the world [83]. Considering the frequent detection of Cipro and its possible negative effects on aquatic organisms and human health, the European Commission has included the antibiotic on the updated Watch List under the Water Framework Directive [84]. It is well known that ciprofloxacin is prone to photochemical transformation by exposure to UV light. According to the literature, the photolysis of Cipro leads to the photosubstitution of fluorine and decarboxylation and the transformation of the piperazine ring [85]. The UV-Vis absorbance spectrum of Cipro at different irradiation times in the absence and presence of the nanostructured ZnO (PTVA) photocatalyst are shown in Figure 8a,b. The band and the humps located at 272 nm and 327 nm gradually decrease with an increase in irradiation time and are almost flattened after 240 min, for the flower-like, nanostructured ZnO (PTVA) coating. As can be seen in Figure 8c, the efficiency of photolysis was enhanced when UV irradiation was combined with the nanostructured ZnO coating. About 96% of the Cipro was degraded under 240 min of UV irradiation, which is 1.5 times larger than the value obtained in the absence of photocatalyst. Furthermore, after 240 min of UV irradiation, the estimated degradation rate of the Cipro over the ZnO sample was 0.0148 min^−1^, which is almost three times larger than the value of bare Cipro (Figure 8d). These results are superior compared to the values obtained for an earlier reported ZnO-based photosystem [86].

**Figure 9:**
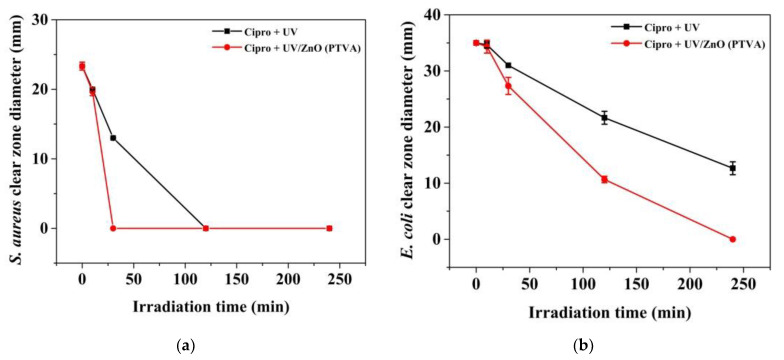
The inhibition-zone diameter of Cipro before and after irradiation in absence or presence of photocatalyst measured for Staphylococcus aureus ATCC 25923 (**a**) and *Escherichia coli* ATCC 8739 (**b**).

Moreover, to assess the photodegradation efficiency of the ZnO coating (PTVA), under UV light for effective treatment of the Cipro present in water, we have performed the agar well-diffusion assay against both Escherichia coli ATCC 8739 (Gram-negative bacilli) and Staphylococcus aureus ATCC 25923 (Gram-positive cocci). Briefly, the antibacterial activity of ciprofloxacin before and after UV irradiation in the absence or presence of photocatalyst has been evaluated. Figure S2 (Supplementary Materials) shows that a clear, hollow inhibition zone is formed on the surface of agar plates containing untreated Cipro solutions, indicating that the bacteria cannot proliferate. In addition, a larger inhibition zone has been measured for the *E. coli* bacteria. The antibacterial study revealed that after photolysis or photocatalytic treatment, the Cipro solution became less toxic, showing a significant decrease in the inhibition zone diameter. Notably, after 30 min of UV irradiation in the presence of nanostructured ZnO (PTVA) coating, the ciprofloxacin lost its antibacterial activity against S. aureus, while for only UV treatment no antibacterial activity is observed around the disc after 120 min (Figure 9a). For the *E. coli* strain, the Cipro completely lost its activity after 240 min of UV irradiation in combination with ZnO photocatalyst, while after 240 min of UV treatment a smaller inhibition zone can still be observed (Figure 9b). These findings imply that the loss of the Cipro’s antibacterial activity is proportional to the time of its UV irradiation, and the nanostructured ZnO (PTVA) coating showed enhancing results towards the photocatalytic removal of antibiotic. Furthermore, microbiological assay proved that the antibiotic degrades completely and that during the photocatalysis process no harmful secondary compounds are generated.

**Figure 10:**
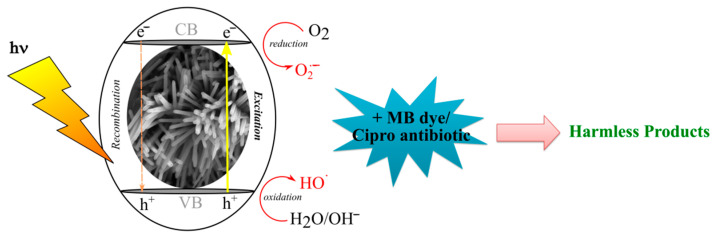
Schematic illustration of the degradation of organic contaminants in the presence of ZnO (PTVA) catalyst.

A possible photocatalytic degradation mechanism of MB and Cipro by the ZnO (PTVA) coating has also been proposed based on previous reports [27,29,39,86,87] and the aforementioned results (Figure 10). First, when nanostructured ZnO coatings are irradiated by UV light with energy *hν* equal to or higher than that of the band gap, electrons (e^−^) in the valence band (VB) may be excited to the conduction band (CB) with the simultaneous generation of holes (h^+^) in the VB. Then, these species (e^−/^h^+^) will react with water molecules and/or hydroxide anions (OH^−^) and oxygen to produce hydroxyl radicals (^•^OH) and superoxide radical anions (O_2_^•−^), respectively. Finally, these radicals may react with MB and Cipro molecules, breaking them into smaller and harmless fragments. The superior photocatalytic activity of flower-like, nanostructured ZnO synthesized by PTVA might be attributed to the synergetic effect between the coating morphology and crystalline order. A high crystalline quality and the high effective surface area of the ZnO nanorods allow for better transport and separation of the photo-excited electrons and holes on different crystal facets, leading to a low recombination rate and enhanced photocatalytic activity.

Compared with the previously available reports (Table 1), the efficiency of our nanostructured ZnO-based photocatalyst synthesized by pulsed TVA is superior for the remediation of water polluted with organic dyes and pharmaceutical compounds. In addition, our photocatalytic system is a valuable alternative to the existing approaches based on particles or powders, owing to the fact that the need for costly and time-consuming post-treatment steps to recover or to remove the suspended particles from the treated water bodies is avoided. As few reports are available for the photocatalytic degradation of organic contaminants from water by using ZnO films [87,88,89,90], tremendous attention has been paid to nanoparticles and powders. For example, films composed of ZnO-porous nanosheets have been synthesized via a hydrothermal approach directly on Zn foil, with improved photocatalytic activity in the degradation of Rhodamine B under UV light irradiation, by Wang and co-workers [88]. Islam et al. assessed the photocatalytic activity of nanostructured ZnO film prepared by a sol–gel dip-coating technique through the decomposition of MB dye [87]. Similarly, the porous ZnO thin coatings prepared by a sol–gel process have been found to be an effective catalyst for the decomposition of chemical waste, such as phenol, chlorophenol, naphthalene and anthracene, to CO_2_ [89]. Photocatalytic activity of ZnO thin films grown by metal–organic chemical-vapour deposition was studied by measuring the photoinduced decolouration of Orange II dye solution [90]. To our best knowledge, this is the first study in which TVA working in a pulsed mode (PTVA) is employed to deposit Zn thin film, and flower-like, nanostructured ZnO (PTVA) coating was used for the successful treatment of ciprofloxacin from water under UV irradiation.

Preliminary results indicate that ZnO (PTVA) coating shows very good photocatalytic activity and stability for water splitting under sunlight irradiation. The photo-electrochemical measurements performed in a conventional three-electrode electrochemical-cell setup with aqueous electrolyte solution (0.1 M NaOH), under standard solar-illumination conditions (AM 1.5 G, 100 mW/cm^2^), reveal that the photocurrent density reaches a value up to 1.5 mA/cm^2^. The photo-electrochemical response for water splitting is a subject of further experimental work, which will be reported on in a separate paper.

## 4. Conclusions

In this work, the Thermionic Vacuum Arc (TVA) deposition technique, operated in DC and a pulsed mode, has been used to synthesize Zn coatings. The thermal annealing/oxidation of Zn coatings leads to the formation of ZnO crystalline nanostructures. The pulsed-plasma regime and higher kinetic energy of the Zn ions gained during PTVA discharge led to the growth of self-assembly nanostructures with a high crystalline order. After thermal annealing in an oxygen atmosphere, the Zn nanostructures synthesized by PTVA change their morphology into nanorods structures, with hexagonal cross-section and high aspect ratio. The effects of morphology and the crystalline facet of ZnO nanostructures on UV-light photocatalytic-decomposition performances of MB dye and the ciprofloxacin antibiotic have been investigated. The degradation efficiency of MB over ZnO photocatalyst synthesized by PTVA reaches 97%, after 60 min of UV irradiation, whereas the decomposition rate constant is nine times higher than ZnO synthesized by DC-TVA. The degradation efficiency of ciprofloxacin over ZnO photocatalyst synthesized by PTVA reaches 88%, after 120 min of UV irradiation. The antibacterial study revealed that ciprofloxacin is removed successfully from water by the UV/ZnO (PTVA) photocatalytic system, without formation of secondary hazardous products. The pulsed TVA deposition of Zn is a favourable and facile method for fabrication of one-dimensional (1D) ZnO nanorods structures, with outstanding photocatalytic performance towards UV photo-degradation of organic contaminants. The superior photocatalytic activity of flower-like, nanostructured ZnO synthesized by pulsed TVA might be attributed to the high crystalline order, high specific surface area and better separation of photo-excited electrons and holes on different crystal facets. On the other hand, the nanostructured ZnO (PTVA) coating, due to its remarkable properties, seems to be an excellent candidate for other applications, such as solar cells and H_2_ generation via solar water splitting.

## Figures and Tables

**Table 1:** Comparative data of photocatalytic degradation of MB dye and antibiotic using ZnO-based photocatalysts.

Catalyst (Amount)	Preparation Method	Contaminants (Concentration)	Light Source	Efficiency/Irradiation Time	k (× 10^−3^, min^−1^)	Ref.
ZnO NPs (0.5 g/L)	Chemical route	MB (10 μM)	300 W Xe lamp	90%/240 min	-	[26]
ZnO NPs (0.02 g/L)		Cipro (15 μM)	UV light (365 nm)	50%/60 min	4.3	[86]
ZnO NPs (0.25 g/L)	Precipitation	MB (63 μM)	UV lamp (Philips, 12 W)	81%/180 min	8.4	[39]
	Sol–gel			92.5%/180 min	12.4	
Mesoporous ZnO (1 g/L)	Sol–gel	MB (20 μM)	250 W Hg lamp	69%/180 min	6.1	[29]
ZnO nanopowder		MB (30 μM, pH = 2)	150 W Hg lamp	86%/180 min	10.8	[91]
ZnO NPs (2.4 g/L)		MB (47 μM)	100 W	85/180 min	12.9	[27]
ZnO film (1 × 1.5 cm^2^)		MB (25 μM)	15 W UV light	60%/380 min	1	[87]
ZnO nanopowder		Cipro	140 W/m^2^	86.9%/75 min	-	[92]
ZnO NPs (0.24 g/L)	Green synthesis	MB (30 μM)	125 W Hg lamp	85%/120 min	17.5	[31]
ZnO NPs		MB (1 mM)	365 nm	63%/120 min	8.12	[93]
ZnO NPs (1 g/L)		MB (47 μM)	10 W Hg lamp	90%/120 min	22.6	[94]
ZnO nanocrystals (0.15 g/L)		MB (16–63 μM)	UV	from 99 to 58%/100 min	-	[95]
Nanostructured ZnO coatings (2 × 2 cm^2^)	DC-TVA	MB (47 μM)	UV lamp (253.7 nm, 1 W/m2)	53%/90 min	6.6	Present work
	PTVA	MB (47 μM)		97%/90 min	58	
		Cipro (0.015 μM)		96%/240 min	14.8	

## Data Availability

Not applicable.

## Supplementary Materials

The following supporting information can be downloaded at: https://www.mdpi.com/article/10.3390/nano12132193/s1, Figure S1: Calibration curves for (a) methylene-blue dye and (b) ciprofloxacin drug; Figure S2: Photographs of Petri dishes used in agar-well diffusion method for both bacterial strains, *E. coli* ATCC 8739 (**a**) and *S. aureus* ATCC 25923 (**b**) and each experimental conditions, where 1 is the control (Cipro), and 2/3, 4/5, 6/7 and 8/9 are Cipro after 10, 30, 120 and 240 min UV exposure in the absence and presence of nanostructured ZnO (PTVA) coatings, respectively. **Calculation of AQY**. Reference [96] is cited in the Supplementary Materials.
