# Supplementary material for: Efficient Removal of Methylene Blue and Ciprofloxacin from Aqueous Solution Using Flower-like, Nanostructured ZnO Coating under UV Irradiation
Source: Nanomaterials (Basel). 2022 Jun 26;12(13):2193. doi: 10.3390/nano12132193 (PMC9267983; doi:10.3390/nano12132193)
Supplement: Supplementary file 1 [file nanomaterials-12-02193-s001.zip › nanomaterials-1785875-supplementary.pdf]

*nanomaterials*

*Supplementary Materials*

# Efficient removal of methylene blue and ciprofloxacin from aqueous solution using flower-like nanostructured ZnO coating under UV irradiation

**Vasile Tiron [1], Mihai Alexandru Ciolan [1], Georgiana Bulai [2], Gabriela Mihalache [2,3], Florin Daniel Lipsa [4] and Roxana Jijie [5]***

[1] Research Center on Advanced Materials and Technologies, Department of Exact and Natural Sciences, Institute of Interdisciplinary Research, Alexandru Ioan Cuza University of Iasi, Iasi 700506, Romania; vasile.tiron@uaic.ro; mihai.ciolan@uaic.ro

[2] Integrated Center of Environmental Science Studies in the North-Eastern Development Region (CERNESIM), Department of Exact and Natural Sciences, Institute of Interdisciplinary Research, Alexandru Ioan Cuza University of Iasi, Iasi 700506, Romania; georgiana.bulai@uaic.ro; gabriela.mihalache@uaic.ro

[3] Department of Horticultural Technologies, "Ion Ionescu de la Brad" University of Life Sciences, M. Sadoveanu Alley, No.3, Iasi, 700490, Romania

[4] Faculty of Agriculture, "Ion Ionescu de la Brad" University of Life Sciences, M. Sadoveanu Alley, No.3, Iasi, 700490, Romania; flipsa@uaiasi.ro

[5] Department of Exact and Natural Sciences, Institute of Interdisciplinary Research, Alexandru Ioan Cuza University of Iasi, Iasi 700506, Romania;

* Correspondence: roxana.jijie@uaic.ro

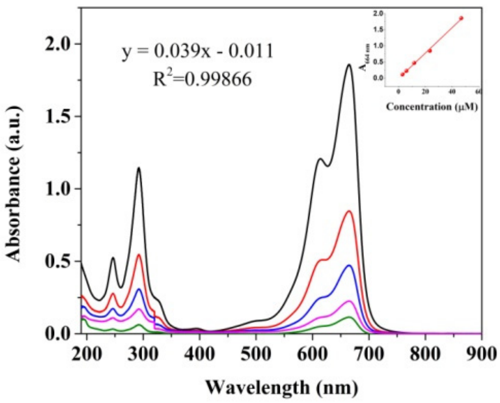


(a)

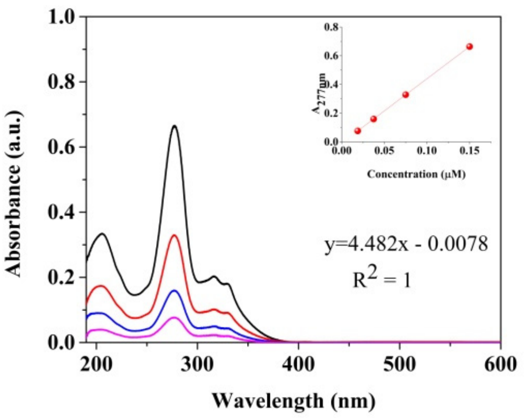


(b)

**Figure S1.** Calibration curves for (a) methylene blue dye and (b) ciprofloxacin drug.

**Copyright:** © 2022 by the authors. Licensee MDPI, Basel, Switzerland. This article is an open access article distributed under the terms and conditions of the Creative Commons Attribution (CC BY) license (https://creativecommons.org/licenses/by/4.0/).

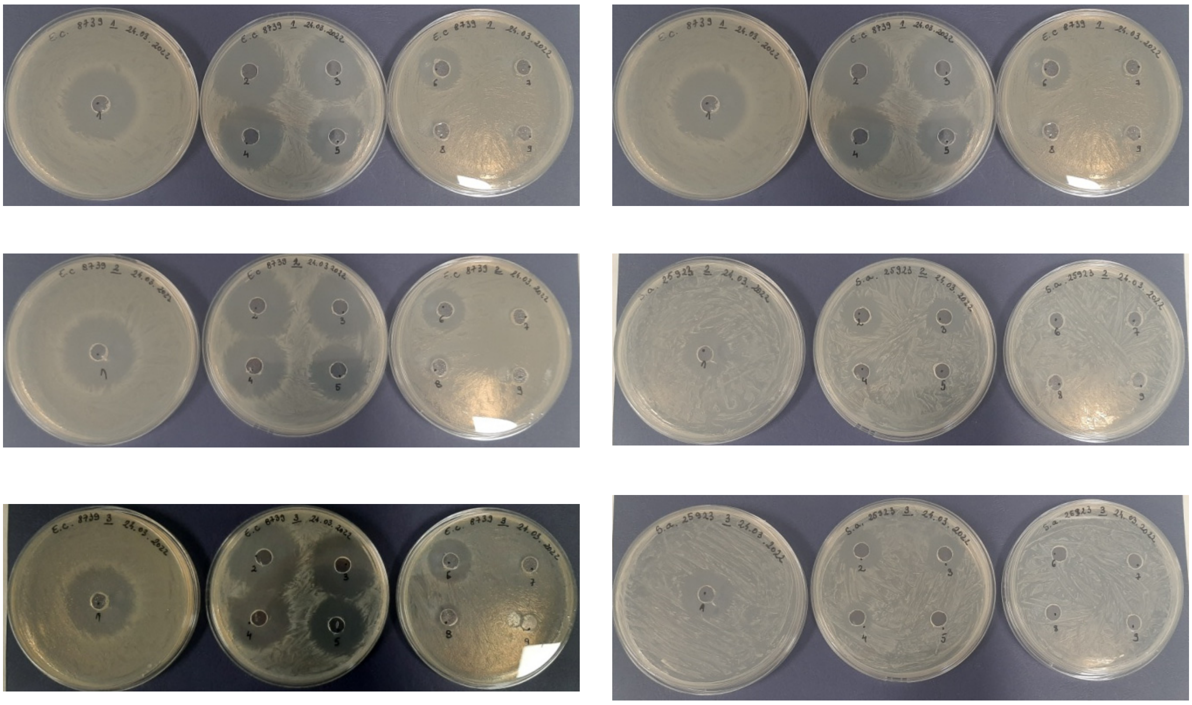

(**a**) (**b**)

**Figure S**2. Photographs of Petri dishes used in agar-well diffusion method for both bacterial strains, *E. coli* ATCC 8739 (a) and *S. aureus* ATCC 25923 (b) and each experimental conditions, where 1 is the control (Cipro), 2/3, 4/5, 6/7 and 8/9 are Cipro after 10, 30, 120 and 240 min UV exposure in the absence and presence of nanostructured ZnO (PTVA) coatings.

**Calculation of AQY**

AQY is defined as the number of reacted electrons occurring per number of incident photons by the system at 253.7 nm, according to Qureshi and Takanabe [96].

AQY (%)= [number of reacted electrons] / [number of incident photons] × 100%

The number of reacted electrons is estimated following the calculation details given by Bora et al. [55].

$$\text{Number of reacted electrons} = \begin{bmatrix}\text{number of electrons requires} \\ \text{for reduction of MB to LMB}\end{bmatrix} \times [n_{MB}(\text{mol})] \times [N_A(\text{mol}^{-1})]$$

where, nMB is the amount of MB molecules degraded in 1800 s, NA is Avogadro's constant.

The number of reacted electrons is determined by the following equation:

$$\text{Number of incident photons} = \begin{bmatrix}\text{light absorbed} \\ \backslash\text{by the photocatalyst (W)}\end{bmatrix} / [\text{average photon energy (J)}] \times t(s)$$

where light absorbed by the photocatalyst and average photon energy are defined by

Light absorbed by the photocatalyst = P (W.m-2)×S (m2)

$$\text{Average photon energy} = \frac{\text{h (J.s)} \times \text{c (m.s}^{-1})}{\lambda\ \text{(m)}}$$

where, P is the power density of the incident monochromatic light, S is the photocatalyst area, λ is the wavelength of the incident monochromatic light, h is Planck's constant and c is speed of light.

ZnO (DC-TVA) photocatalyst

Number of reacted electrons =[2] × [$3.3\times10^{-8}$(mol)] × [$6.022\times10^{23}$(mol$^{-1}$)]=3.98×1016

Number of incident photons = 9.19×1017

AQY (%) = 4.3 %

ZnO (PTVA) photocatalyst

Number of reacted electrons =[2] × [$2.05\times10^{-7}$(mol)] × [$6.022\times10^{23}$(mol$^{-1}$)]=2.47×1017

Number of incident photons = 9.19×1017

AQY (%) = 26.9 %
